# Fat Chance? A High-Fat Diet May Offset the Effects of Developmental Neurotoxicity

**DOI:** 10.1289/ehp.117-a257b

**Published:** 2009-06

**Authors:** Angela Spivey

Widespread exposure to a variety of neurotoxic chemicals has been posited as one potential factor behind what has been called a “silent pandemic” of autism spectrum disorders, learning disabilities, and other neurodevelopmental disorders. Exposure to organophosphate insecticides is of particular concern because these widely used compounds have been shown in rodents to induce persistent synaptic abnormalities in neural acetylcholine (ACh) systems at doses too low to cause symptoms of systemic exposure. Pilot studies have reported some evidence of improvement when a “ketogenic” diet—high in fat and low in carbohydrates—was used to treat certain neurologic disorders. Drawing from this preliminary clinical research, researchers have now demonstrated that many of the abnormalities in ACh systems produced by neonatal organophosphate exposure were not evident in adult rats fed a high-fat diet **[*EHP*** **117:916–922; Slotkin et al.]**.

Rats were injected with the organophosphate parathion on each of postnatal days 1–4, at doses of 0.1 or 0.2 mg/kg/day—these dosages straddle the threshold at which cholinesterase inhibition is first detectable. In adulthood, half the animals were switched to a high-fat diet for 8 weeks. The investigators then examined brain regions of the rats to assess specific aspects of ACh synaptic function, including nicotinic ACh receptor binding, choline acetyltransferase activity, and hemicholinium-3 binding to the presynaptic choline transporter.

Adult rats on a standard lab chow diet showed multiple abnormalities in regional ACh synaptic markers following parathion exposure. All seven abnormalities observed in parathion-exposed females on the standard diet were absent in exposed females on the high-fat diet, and eight of ten abnormalities observed in parathion-exposed males on the standard diet were absent in exposed males on the high-fat diet. The results suggest that diet may offer a way to ameliorate the effects of developmental neurotoxicant exposure.

However, the authors offer several caveats. Their earlier work showed that neonatal exposure to organophosphates produced long-term changes in metabolic function that have been linked with obesity, prediabetes, and cardiovascular risk factors such as elevated serum lipids. Because these metabolic abnormalities could be exacerbated by a high-fat diet, future studies should seek to uncover whether and how specific aspects of the diet influence abnormalities in ACh systems. Moreover, although this study showed that dietary modifications may offset synaptic changes, future studies will need to determine whether such modifications can actually lead to improved neurobehavioral outcomes.

The authors also highlight a potential connection between early-life toxicant exposure and subsequent diet-related disease. If a high-fat diet can indeed ameliorate the impact of developmental neurotoxicants, then this might serve as an underlying, subconscious reinforcement to consume a high-fat diet as a way of self-remediating underlying neurobehavioral deficits—potentially expanding the public health implications of the developmental effects of neurotoxicant exposure.

